# Mechanisms of oxysterol-induced carcinogenesis

**DOI:** 10.1186/1476-511X-10-44

**Published:** 2011-03-09

**Authors:** Apinya Jusakul, Puangrat Yongvanit, Watcharin Loilome, Nisana Namwat, Rahul Kuver

**Affiliations:** 1 Department of Biochemistry, Faculty of Medicine, Khon Kaen University, Khon Khaen 40002, Thailand; 2 Liver Fluke and Cholangiocarcinoma Research Center, Faculty of Medicine, Khon Kaen University, Khon Kaen 40002, Thailand; 3 Department of Medicine, Division of Gastroenterology, University of Washington School of Medicine and the Puget Sound Veterans Affairs Health Care System, Seattle, WA, USA

## Abstract

Oxysterols are oxidation products of cholesterol that are generated by enzymatic reactions mediated by cytochrome P450 family enzymes or by non-enzymatic reactions involving reactive oxygen and nitrogen species. Oxysterols play various regulatory roles in normal cellular processes such as cholesterol homeostasis by acting as intermediates in cholesterol catabolism. Pathological effects of oxysterols have also been described, and various reports have implicated oxysterols in several disease states, including atherosclerosis, neurological disease, and cancer. Numerous studies show that oxysterols are associated with various types of cancer, including cancers of the colon, lung, skin, breast and bile ducts. The molecular mechanisms whereby oxysterols contribute to the initiation and progression of cancer are an area of active investigation. This review focuses on the current state of knowledge regarding the role of oxysterols in carcinogenesis. Mutagenicity of oxysterols has been described in both nuclear and mitochondrial DNA. Certain oxysterols such as cholesterol-epoxide and cholestanetriol have been shown to be mutagenic and genotoxic. Oxysterols possess pro-oxidative and pro-inflammatory properties that can contribute to carcinogenesis. Oxysterols can induce the production of inflammatory cytokines such as interleukin-8 and interleukin-1β. Certain oxysterols are also involved in the induction of cyclo-oxygenase-2 expression. Inflammatory effects can also be mediated through the activation of liver-X-receptor, a nuclear receptor for oxysterols. Thus, several distinct molecular mechanisms have been described showing that oxysterols contribute to the initiation and progression of cancers arising in various organ systems.

## Introduction

**Figure 1:**
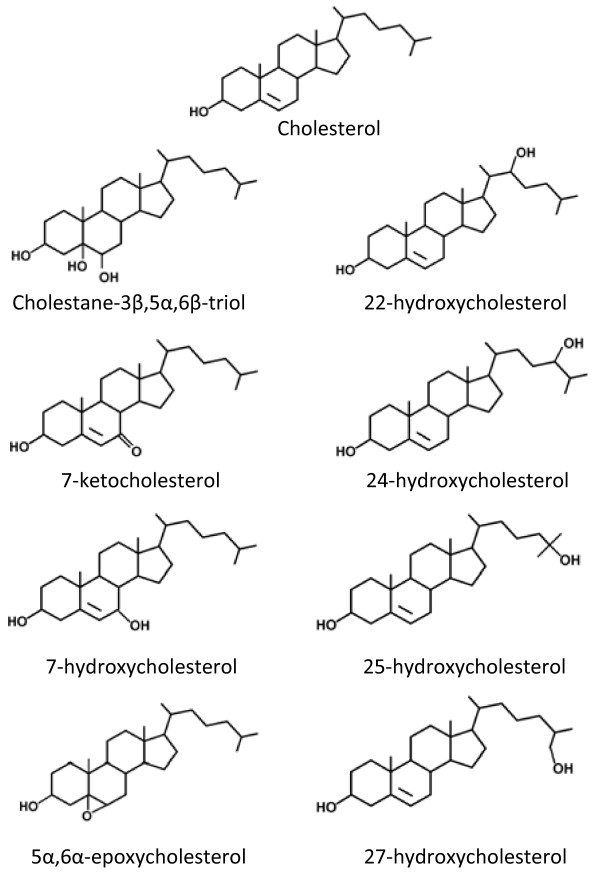
**Structure of cholesterol and several common oxysterols**.

Oxysterols are derived from either enzymatic or non-enzymatic oxidation of cholesterol [1,2]. The chemical structures of oxysterols vary depending upon the number and position of oxygenated functional groups, and include keto-, hydroxyperoxy-, and epoxy forms (Figure 1). Enzymatic pathways of oxysterol production mainly involve cytochrome P450 family enzymes. Examples include 27-hydroxylase (CYP27A1), the key enzyme in the alternative bile acid synthesis pathway which leads to the production of 27-hydroxycholesterol [3,4]; and 7α-hydroxylase (CYP7A), the rate limiting enzyme in the classical bile acid synthesis pathway that leads to the production of 7α-hydroxycholesterol [3,5]. Certain oxysterols are produced by non-enzymatic oxidation (or auto-oxidation), a process that involves reactive oxygen and nitrogen species (ROS, RNS). Examples of ROS that participate in non-enzymatic oxidation of cholesterol include hydroxyl radical (^.^OH), hydrogen peroxide (H_2_O_2_), singlet oxygen (^1^O_2_) and ozone (O_3_) [6,7]. 7α-hydroperoxycholesterol is an example of a product of non-enzymatic oxidation of cholesterol which can be further oxidized by non-enzymatic and enzymatic means. Examples of oxysterols generated by this mechanism are 7-ketocholesterol and 7α/β-hydroxycholesterol.

Numerous *in vivo *and *in vitro *studies have described biological effects of oxysterols [5]. Oxysterols can act as intermediates in cholesterol catabolism, especially in bile acid synthesis and are therefore involved in the elimination of excess cholesterol from the body. As oxysterols possess more hydrophilic moieties compared to the parent cholesterol molecule, they can pass more easily through cell membranes. This can occur because polar moieties inserting into hydrophobic regions of cell membranes result in redistribution of the sterol in conjunction with local re-ordering of the acyl chains [3,8,9]. Oxysterols are also able to regulate key enzymes in cholesterol turnover such as 3-hydroxy-3-methylglutaryl (HMG)-coenzyme A (CoA) reductase [10], the rate-limiting enzyme in cholesterol synthesis.

Pathogenic effects of oxysterols has been described predominantly in cardiovascular diseases [6,11,12] and degenerative diseases such as age-related macular degeneration [13], Alzheimer's disease [14,15] and osteoporosis [16]. Several reports report a role for oxysterols in neurological disease (summarized in Table 1) [17-20].

**Table 1:** Oxysterol disease associations

Oxysterols	Cancer	Atherosclerosis	Neurological diseases	References
5,6-cholesterolepoxide	√	√		[84,85]
Cholestane-3β,5α,6β-triol	√	√		[17,86,87]
25-hydroxycholesterol			√	[88]
24-hydroxycholesterol			√	[89-91]
7-Hydroperoxy- cholesterols		√		[92]
7-hydroxycholesterol		√		[93]

The effects of cholesterol oxidation products on carcinogenesis was first reported by Bischoff and Rupp in 1946 [21]. Ovariectomized mice that received 10 mg of crude progesterone in sesame oil showed a higher incidence of cancer than mice that received purified progesterone. Cholesterol oxidation products were found in mice that received the crude progesterone extract. This study provided circumstantial evidence that carcinogens may be produced endogenously from cholesterol. Subsequent studies have demonstrated the carcinogenic potential of specific oxysterols. For example, 6β-hydroperoxy-4-cholestane-3-one injected subcutaneously led to the development of local sarcomas in mice [22]. Cholesterol-5α, 6α-epoxide also induced local sarcomas [23].

### Role of oxysterols in tissue-specific carcinogenesis

#### Oxysterols and lung cancer

An epidemiological study described the relationship between lung cancer risk and oxysterols. Using a nested case-control study design, smoking-independent associations between cholesterol oxidation and lung cancer risk were investigated. Six oxysterols (7α-hydroxycholesterol, 7β-hydroxycholesterol, cholesterol-α-epoxide, cholesterol-β-epoxide, cholestanetriol, 7-ketocholesterol and 7-keto-pregnenolone), were measured in plasma of 20 lung cancer patients. The results showed slightly increased levels of all oxysterols compared with control with the exception of 7α-hydroxycholesterol and cholesterol-α-epoxide. Additionally, a risk analysis showed no statistical significant link between oxysterol concentrations and lung cancer development. When adjusted for participation in sports, however, the association between 7β-hydroxycholesterol levels and lung cancer was statistically significant. The data suggest that plasma levels of 7β-hydroxycholesterol are a smoking-independent predictor and biomarker for lung cancer risk [24]. Increased levels of 7β-hydroxycholesterol may be caused by lipid peroxidation, oxysterol dietary intake and individual differences in the metabolism of oxysterols.

Several reports describe the anti-cancer effects of 7β-hydroxycholesterol in lung cancer and various other types of cancer [25-28]. 7β-hydroxycholesterol mediated a cytotoxic effect on human NCI-H460 lung cancer cells through the induction of apoptosis via caspase activation [29].

#### Role of oxysterols in colon carcinogenesis

Several reports have provided evidence that oxysterols contribute to gastrointestinal cancer development. Animal fat oxidation products, including oxysterols, have been linked to colon carcinogenesis. One possible mechanism for this effect is via upregulation of expression of transforming growth factor-β1 (TGF-β1) in macrophages and fibroblasts, cells which are present in the tumor microenvironment [30]. TGF-β1 is normally involved in promoting differentiation and apoptosis in intestinal epithelial cells. Colon cancer cells demonstrate decreased expression of TGF-β1 type I and/or type II receptors [31]. Thus, colon cancer cells are less susceptible to the growth regulatory effects of TGF-β1. An increased concentration of TGF-β1 is thought to be involved in a selective elimination of neoplastic cells susceptible to the pro-apoptotic effects of this cytokine, thereby indirectly allowing the clonal expansion of TGF-β1-insensitive neoplastic cells [32,33]. Consequently, disruption of TGF-β1 growth inhibition would support uncontrolled cell proliferation and transformation. Such alterations in TGF-β1 receptor expression have been described in cancers of the colon [34], prostate [35], breast [36] and lung [37]. An oxysterol mixture composed of 7α-hydroxycholesterol (43%), 5α, 6α-epoxycholesterol (32%) and 5β, 6β-epoxycholesterol (6%) induced apoptosis in the human colon adenocarcinoma cell line CaCo-2. The mitochondrial pathway of apoptosis was upregulated in these cells after stimulation by oxysterols as shown by a decrease in mitochondrial membrane potential, release of cytochrome c, and activation of caspase-3. The mechanism of oxysterol-induced upregulation of apoptosis in differentiated CaCo-2 cells involved increased production of ROS by NADPH oxidase activation [38]. Excessive levels of ROS and pro-inflammatory cytokines likely contribute to impairment of enteric mucosal function [39].

Patients with ulcerative colitis are at risk for developing colon cancer. These patients excrete high levels of cholesterol, coprostanol and cholestane-3β-5α-6β triol (cholestanetriol) in feces compared to patients with other digestive diseases and healthy controls [40]. However, the carcinogenic effect of oxysterols has not been demonstrated in rat colons. No colonic tumors were induced by sterols including cholesterol epoxide, cholestanetriol and cholesterol in standard and germ free female rats. Only lithocolic acid, a bile acid produced by bacterial oxidation of chenodeoxycholic acid in the colon, induced colon cancer in this model [41].

#### Role of oxysterols in cholangiocarcinogenesis

Oxysterols may also play a role in the development and progression of cholangiocarcinoma (CCA), a malignancy arising from intra- and extra-hepatic bile duct epithelial cells or cholangiocytes. A risk factor for the development of CCA in Thailand is chronic infection with the liver fluke, *Opisthorchis viverrini *[42,43]. Infection leads to accumulation of nitric oxide (NO) and oxygen radicals in inflamed tissue resulting in direct and indirect DNA damage both in animal and human studies [44-47]. We found significantly increased levels of 4-cholesten-3-one and cholestanetriol in a hamster model of CCA induced by liver fluke infection combined with *N*-nitrosodimethylamine treatment (unpublished data). Interleukin 6 (IL-6) is a potent mitogen for cholangiocytes and cholangiocarcinoma cells [48-50]. The role of oxysterols in mediating tumor formation in cholangiocytes was reported by Yoon et. al.[51]. These investigators examined the effect of 22(R)-hydroxycholesterol (22-HC) on cyclooxygenase-2 (COX-2) expression and found that this oxysterol stabilized the level of COX-2 mRNA via a p38-mitogen activated protein kinase (MAPK)-dependent mechanism leading to COX-2 protein accumulation in a CCA cell line. This induction of COX-2 could promote cellular replication and inhibit apoptosis [52] and thus contribute to the progression of CCA. In addition to induction of COX-2 expression, bile acids also enhance cellular levels of a potent anti-apoptotic protein, myeloid cell leukemia protein 1 (Mcl-1) [53]. Given the important role of apoptosis inhibition in carcinogenesis and the dominant anti-apoptotic effects of Mcl-1 in CCA cells, this observation may be important in understanding the tropism displayed by bile acid-induced CCA. Indeed, CCA appears to have co-opted the toxic bile milieu to promote growth and survival. Alteration of bile composition during chronic inflammation warrants further investigation as a mechanism of biliary tract carcinogenesis. This observation suggests that these tumors may not only have developed mechanisms to survive the toxic constituents in bile but may actually use bile to promote growth and survival.

Oxysterols were found in infected human hepatic bile, with 7α-hydroxycholesterol and 7β-hydroxycholesterol present at significantly higher concentrations in infected than in uninfected bile. The levels of 7α-hydroxycholesterol, 7β-hydroxycholesterol, cholestanetriol, 7-α-hydroxy-4-cholestane-3-one and 7-ketocholesterol were also positively correlated with C-reactive protein (CRP) levels. These findings suggest that infection of biliary cells may be linked to the synthesis of oxysterols and consequently participate in the development of biliary diseases [54,55].

### Mechanisms of oxysterols in carcinogenesis

#### Mutagenicity of oxysterols

The mutagenic effects of oxysterols were first described in1979. Smith *et al. *[56] reported this effect in air-aged samples of cholesterol on five strains of *Salmonella typhimurium *using the Ames method. Exposure of pure cholesterol to heating in air at 70°C or to 60°C or γ-radiation resulted in the genesis of mutagenic compounds. No mutagenic effect of cholesterol-α-epoxide and cholesterol-β-epoxide was shown, similar to cholesterol. In a hamster model, cholesterol-α-epoxide showed a weak mutagenic effect. V79 Chinese hamster lung fibroblasts treated with cholesterol-α-epoxide induced 8-azaguanine-resistant mutants at frequencies that were 4.6 to 11.8-fold higher than the spontaneous mutation rate in rats. Cholesterol-α-epoxide accumulates in cells and can transform into cholestanetriol which possesses greater cytotoxicity and acts as a potent inhibitor of DNA synthesis. This modification causes a decrease in the mutagenic effects of cholesterol-α-epoxide. Thus the mutagenicity of cholesterol-α-epoxide in V79 Chinese hamster lung fibroblast cells may depend on the ability of cell to metabolize cholesterol-epoxide to cholestanetriol via epoxide hydrolase activity.

The genotoxicity of cholestanetriol using the Ames method and the generation of chromosomal aberrations has been demonstrated. Cholestanetriol, but not 7-ketocholesterol and cholesterol-5α-6α-epoxide, exhibited genotoxicity as determined by the rate of bacterial revertants in the absence of the metabolic activating enzyme (S9). In addition, cholestanetriol induced chromosomal aberrations in Chinese hamster ovary cells (CHO-K1) in the presence and absence of the S9 fraction. However, the degree of abnormalities in chromosomal structure was reduced with S9 treatment when compared to no treatment. Anti-oxidant enzymes including catalase and superoxide dismutase can inhibit cholestanetriol-induced increases in bacterial revertant rates and chromosome aberration in CHO-K1 cells. ROS production also increased in cholestanetriol-treated cells. These results suggest that genotoxicity of cholestanetriol may involve ROS generation [57]. Moreover, the effect of oxysterols on DNA mutation was reported by Gramajo et al.[58]. 7-ketocholesterol-treated culture significantly increased the degree of mitochondrial DNA (mtDNA) damage in the human RPE cell line (ARPE-19). Various studies also provide evidence that oxysterols enhance the production of ROS/RNS [57-62]. Oxysterols might enhance the production of ROS/RNS through alterations in the mitochondrial electron transport chain [59].

#### Role of oxysterols in oxidative stress and inflammation

Numerous studies have found a role for oxysterols in mediating pro-oxidative and pro-inflammatory processes in age-related degenerative diseases such as Alzheimer's disease and atherosclerosis [6,13,63,64]. The pro-oxidative and pro-inflammatory effect of 7α-, 7β-hydroxycholesterol, 7-ketocholesterol, cholesterol-5α-6α-epoxide, cholesterol-β-epoxide, 22R-, 22S-, 25- and 27-hydroxycholesterol were studied in U937 human promonocytic leukemia cells. 7β-hydroxycholesterol, 7-ketocholesterol and cholesterol-β-epoxide increased the production of singlet oxygen. The highest capacity to stimulate IL-8 secretion and enhance IL-8 mRNA levels was observed in cells treated with 7β-hydroxycholesterol and 25-hydroxycholesterol [18]. Oxysterols that were oxidized at C7 enhanced the production of free radicals [62,65,66]. 7-ketocholesterol caused a decrease in cellular glutathione content, oxidation of polyunsaturated fatty acids and enhanced the production of ROS [62]. Moreover, 7-ketocholesterol enhanced IL-1β secretion in endothelial cells and participated in the recruitment of monocytes and T lymphocytes.

The cytotoxicity of 7-ketocholesterol and 7β-hydroxycholesterol involves induction of oxidative stress and down-regulation of anti-oxidative mechanisms [62]. These effects can be inhibited by anti-oxidant compounds such as lycopene, carotene, folate, glutathione, N-acetylcysteine and resveratrol [64,65,67-70]. Notably, the pro-oxidative and cytotoxic effects may occur in the absence of activation of NADPH oxidase, as shown in J774A.1 macrophage cells [60,71]. NADPH oxidase plays a key role in oxysterol-induced ROS production in a human colon adenocarcinoma cell line [38].

Pro-inflammatory effects of oxysterols have also been described. 7-ketocholesterol, 7β-hydroxycholesterol, 24-hydroxycholesterol, 25-hydroxycholesterol and cholestanetriol have the capability to induce IL-8 production. The pro-inflammatory effects of 25-hydroxycholesterol was increased in hypoxic culture conditions and potentiated lipopolysaccharide (LPS)-induced IL-1β secretion [72]. In U937 and THP-1 cells, 7β-hydroxycholesterol and 25-hydroxycholesterol were involved in the recruitment of immunocompetent cells via MCP-1, MIP-1β, TNF-α, IL-1β and IL-8 [73]. In addition, certain oxysterols are involved in the induction of COX-2 expression which correlates with CRP levels [51,55].

Pro-inflammatory effects of oxysterols also act at the transcriptional level. This can involve the activation of the liver-X-receptor (LXR) which is a nuclear receptor for certain oxysterols [74]. Pro-inflammatory oxysterols that exert their effect through LXR include 22R-hydroxycholesterol and 25-hydroxycholesterol but not 7β-hydroxycholesterol [19]. 7β-hydroxycholesterol appears to exert its pro-inflammatory effects at translational and post-translational levels. In 7β-hydroxycholesterol-treated U937 and THP-1 cells, increased IL-8 secretion was found. This activation is associated with activation of the MEK/ERK1/2 signaling pathway [73]. The anti-inflammatory effects of oxysterols were also described in human macrophages incubated with 7-ketocholesterol, 7β-hydroxycholesterol, 25-hydroxycholesterol and 27-hydroxycholesterol before exposure to LPS-induced TNF-α secretion [75]. The 25-hydroxycholesterol which was produced from the stimulation of macrophage toll-like receptors (TLRs) can suppress IL-2-mediated stimulation of B cell proliferation and block IgA class switch recombination, resulting in decreased IgA production [76].

#### Oxysterol signaling pathways

Oxysterols may exert their cellular effects by binding to specific proteins and activating signaling cascades. In human breast cancer (MCF-7) cells, Silva *et al. *[77] found that oxysterols in the lipid fraction isolated from human osteoblast-like MG63 cell-conditioned medium (MG63CM) induced MCF-7 cell migration to bone tissue. Interestingly, insulin-like growth factor 1 increased the expression of oxysterol binding protein-related protein 7 (ORP7) in MCF-7 cells. Binding between these ligands and receptors altered the organization of lipid microdomains in cell membranes. Several oxysterol-binding proteins such as ORP7 and ORP 3 were recently reported to play a role in cell adhesion and migration through R-Ras, a small GTPase [78]. ORP3 is abundantly expressed in leukocytes and epithelial cells, and abnormally high expression levels are found in leukemias and solid tumors [79]. Oxysterol-binding protein-like-8 is overexpressed in hamster induced cholangiocarcinoma [80].

**Figure 2:**
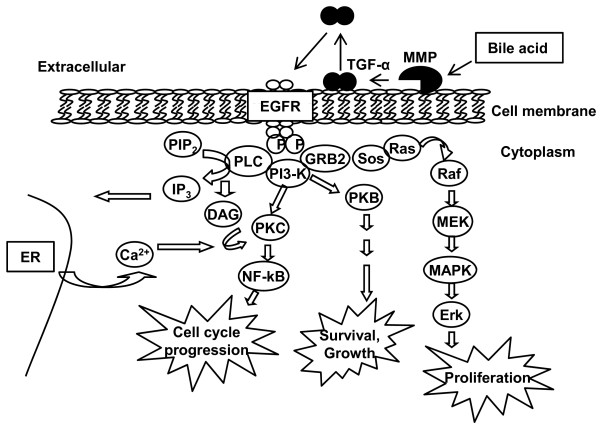
**Bile acids transactivate EGFR in cholangiocytes**. Bile acids induce a signaling transduction cascade through EGFR. Bile acids can stimulate Src kinase activity, enhance MMP activity and generate TGF-α. Soluble TGF-α acts as the ligand for activation of EGFR. This activation induces signaling molecules such as protein kinase C (PKC), PI3-K and MAPK resulting in alterations in the cell cycle, cell growth, and cell survival (adapted from [53].

Oxysterols may act as secondary messengers in signaling transduction pathways. 22R-hydroxycholesterol stabilizes COX-2 mRNA via the 38-MAPK pathway in human cholangiocytes [51]. Hepatocellular carcinoma cells HepG2 and Huh7 senesce via alteration of p38 MAPK when treated with 5,6 secosterol [81]. Moreover, bile acids, which can be considered a type of oxysterol in bile, are known to activate various signaling cascades. Bile acids can stimulate cell proliferation by activation of the phosphatidylinositol-3-kinase (PI3-K) pathway via a number of mitogen receptor tyrosine kinases such as epidermal growth factor receptor (EGFR) [82]. This activation is mediated by TGF-α, which is a ligand for EGFR. Bile acids that transactivate EGFR through a TGF-α dependent mechanism can be blocked by matrix metalloproteinase (MMP) inhibitors. This suggests that bile acids may activate the release of TGF-α through the induction of MMP. The simplest interpretation of this finding is that bile acids induce MMP activity by generating soluble TFG-α leading to activation of EGFR (Figure 2). Rats with obstructed bile ducts demonstrate increased cholangiocyte proliferation and secretion [83].

## Conclusions

**Figure 3:**
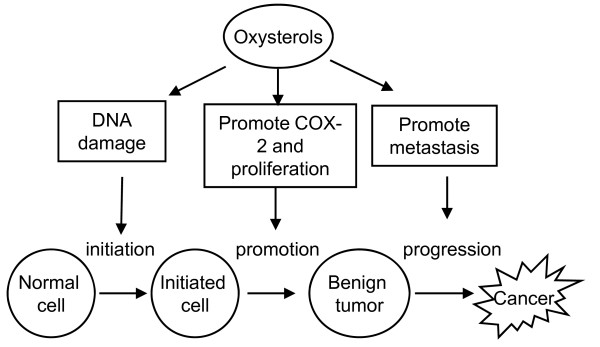
**Effects of oxysterols on carcinogenesis**. Oxysterols exert their effect on three stages of carcinogenesis by induction of DNA damage, enhancing production of COX-2 and stimulation of tumor cell migration [51,57,77].

This review focuses on the mechanisms that may be operative in oxysterol-induced or associated carcinogenesis. *In vitro *and *in vivo *studies have demonstrated an association between different types of oxysterols and the development and progression of cancer of the colon, lung, breast and bile ducts. Oxysterols may play critical roles in multiple stages of carcinogenesis (Figure 3). First, they may be involved in tumor initiation by enhancing the production of ROS/RNS. Second, tumor promotion may be enhanced by oxysterols through upregulated expression of proteins such as COX-2 leading to the alteration of cellular phenotypes. In addition, certain oxysterols can support cancer progression through the induction of migration. Oxysterols may exert their effect by binding to specific proteins and activating signaling cascades. Further investigations of oxysterols and their impact on carcinogenesis are required for developing improved strategies for the prevention and treatment of various types of cancer.

## Competing interests

The authors declare that they have no competing interests.

## Authors' contributions

AJ wrote the manuscript. WL, PY, NN, and RK reviewed and revised the manuscript. All authors approved the final version of the manuscript.
